# Altered baseline brain activity differentiates regional mechanisms subserving biological and psychological alterations in obese men

**DOI:** 10.1038/srep11563

**Published:** 2015-06-23

**Authors:** Bin Zhang, Derun Tian, Chunshui Yu, Meng Li, Yufeng Zang, Yijun Liu, Martin Walter

**Affiliations:** 1 Department of Anatomy, Tianjin Medical University, Tianjin, China; 2 Department of Psychiatry and Psychotherapy, University of Magdeburg, Magdeburg, Germany; 3 Clinical Affective Neuroimaging Laboratory, University of Magdeburg, Magdeburg, Germany; 4 Leibniz Institute for Neurobiology, Magdeburg, Germany; 5 Department of Radiology, Tianjin Medical University General Hospital, Tianjin, China; 6 Center for Cognition and Brain Disorders, Hangzhou Normal University, Hangzhou, China; 7 Key laboratory of Cognition and Personality (SWU), Ministry of Education, Chongqing, China; 8 School of Psychology, Southwest University, Chongqing, China

## Abstract

Obesity as a chronic disease is a major factor for insulin resistance and Type 2 diabetes, which has become a global health problem. In the present study, we used resting state functional MRI to investigate the amplitude of low frequency fluctuations of spontaneous signal during both hunger and satiety states in 20 lean and 20 obese males. We found that, before food intake, obese men had significantly greater baseline activity in the precuneus and lesser activity in dorsal anterior cingulate cortex (dACC) relative to lean subjects. Furthermore, after food intake, obese males had significantly lesser activity in dACC than lean males. We further found a significant positive correlation between precuneus activation and hunger ratings before food intake, while dACC activity was negatively correlated with plasma insulin levels before and after food intake. These results indicated that both precuneus and dACC may play an important role in eating behavior. While precuneus rather seemed to mediate subjective satiety, dACC levels rather reflected indirect measures of glucose utilization.

Obesity, a result of an imbalance of eating behavior and energy expenditure has been associated to numerous general health problems^1^, specifically due to its relationship to insulin resistance which is the major factor leading to Type 2 diabetes^2^.

In general, human eating behavior is modulated by physiological, psychological, and cognitive factors^3^,^4^,^5^. Functional MRI (fMRI), a method to non-invasively investigate the brain as a master organ regulating homeostatic behavior, has been widely applied to the study of feeding behavior. In a study of non-obese subjects by Passamonti^6^, they found that viewing pictures of appetizing food resulted in alterations in functional connectivity along the ventral striatum, anterior cingulate and premotor cortex compared to bland food photos. Recently, there has been increased interest in the altered activity of cerebral networks in regulating eating behavior in the obese individuals^7^,^8^,^9^,^10^,^11^. The default mode network (DMN) and salience network (SN) are considered to be two main target networks, given their crucial roles involving the integration of homeostatic signals and cognitive control. Tregellas and colleges reported altered function of the DMN in obesity individuals^10^. Kullmann and colleges reported that the obese/overweight subjects showed augmented response in the SN towards food stimuli, which indicated a potentially top-down deviancies driving the overconsumption of food in the obese population^7^. A recent study further examined the response for viewing of food and nonfood images in pre-meal and post-meal states. The authors reported increased activation in ACC during the pre-meal condition and greater activation in medial prefrontal cortex in obese subjects in pre-meal and post-meal states. This altered activation in salience network was considered to be associated with the overeating through an imbalance between autonomic processing and award processing of food stimuli^9^.

So far, various brain regions and networks have been shown to be associated with eating behavior, while hunger and satiety signals in related regions seemed abnormal in obese individuals^7^. Functional deficient underlying eating behavior of humans and their changes with food motivation in obese have remains largely unknown. Meanwhile, instead of the functional connectivity, which focuses on the signal temporal synchronization of low frequency fluctuation (LFF) among different brain areas^12^, amplitude of low frequency fluctuations (ALFF) takes the amplitude of brain activity as measured by BOLD signals in resting-state fMRI into account^13^. In ALFF, the power spectrum of BOLD signals in the low-frequency range is used for calculating correlations to estimate the degree of functional connectivity among voxels^13^,^14^. In the present study, we used ALFF to assess altered activities in local baseline brain activity in obese male subjects during hunger and satiety states. The purpose of the study was to find out the core regions which are associated with the obesity in the context of hunger or satiety state. Two major factors (hunger and satiety state) mediating eating behavior were considered separately, disentangling subjective feeling of hunger from objective mechanisms of altered insulin concentration. We expected that these two factors contribute differently in the context of satiety or hunger, while the primary regions of action were to be defined for the two mechanisms.

## Results

We used independent samples *t-*test to compare the group differences of plasma glucose, insulin and the rating of hunger (in the subjects with obesity and lean male subjects). Plasma glucose levels were similar between lean and obese subjects before and after liquid intake. Plasma insulin concentrations of obese subjects were significantly higher (p < 0.05) than lean subjects during the entire process. There were no differences of the rating of hunger between the groups. There further were no significant difference of hunger rating between two groups before and after liquid intake (Table 1).

ANOVA revealed significant main effects of group on dACC (x = −3, y = 9, z = 24, k = 154, Suppl. Fig. 1A) and main effects of feeding condition in pericental lobule (x = −6, y = −24, z = 60, k = 202, Suppl. Fig. 1B). This effect was mainly driven by increases after feeding in controls while in obese individuals, ALFF was significantly increased in PCC (x = 9, y = −33, z = 30, k = 174) after feeding (Suppl. Fig. 2B).

**Figure 1:**
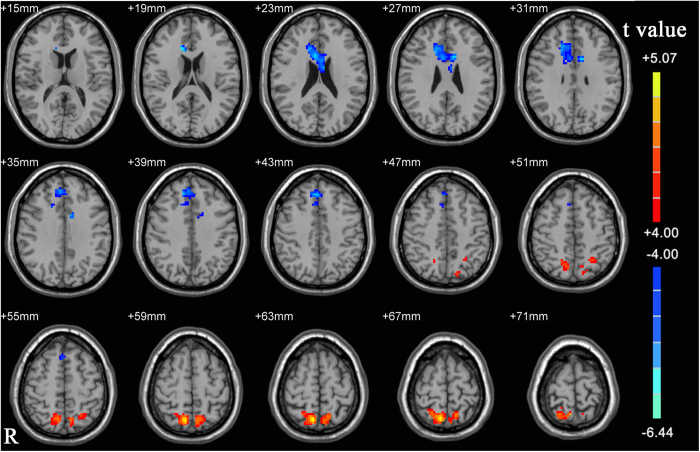
A T-statistic map showed the ALFF differences before liquid intake between obese subjects and controls (p < 0.05, corrected). Hot and cold colors indicate greater and lower ALFF activity in obese subject relative to healthy controls, respectively.

**Figure 2:**
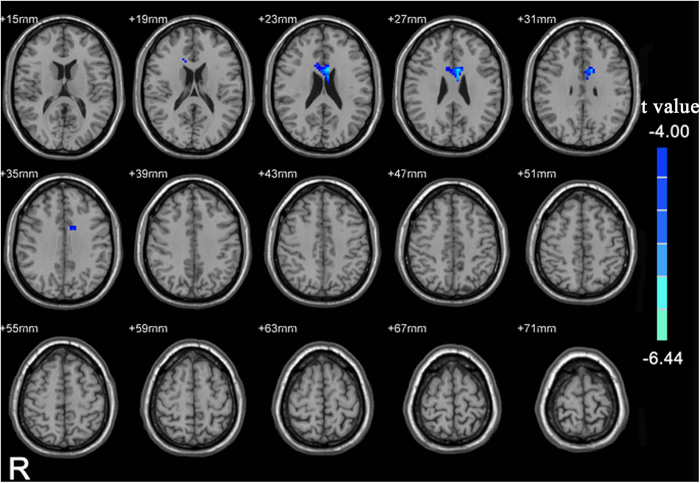
A T-statistic map showed the ALFF differences after liquid intake between obese subjects and controls (p < 0.05, corrected). Cold color indicates lower ALFF activity in obese subject relative to healthy controls.

Before liquid intake, obese male subjects, compared to the lean control group, had significantly greater resting state activity in the bilateral precuneus (Fig. 1, p-value < 0.05, corrected; right precuneus: x = 9, y = −66, z = 60, k = 153; left precuneus: x = −12, y = −60, z = 66, k = 62) and significantly lower resting state activity in the dorsal anterior cingulate cortex (dACC) (Fig. 1, p-value < 0.05, corrected, x = 15, y = 24, z = 18, k = 150). After liquid intake, only the lower activity in the dACC (Fig. 2, p-value < 0.05, corrected, x = −6, y = 9, z = 24, k = 49) compared to the control group, however no increased activity in the precuneus was not observed.

**Figure 3:**
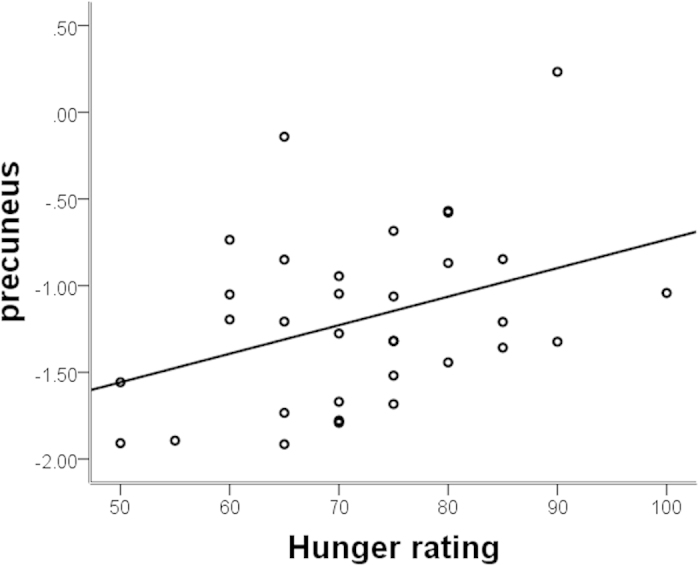
Partial correlation adjusted for BMI between the mean activity of precuneus in the pre-meal condition and hunger ratings.

**Figure 4:**
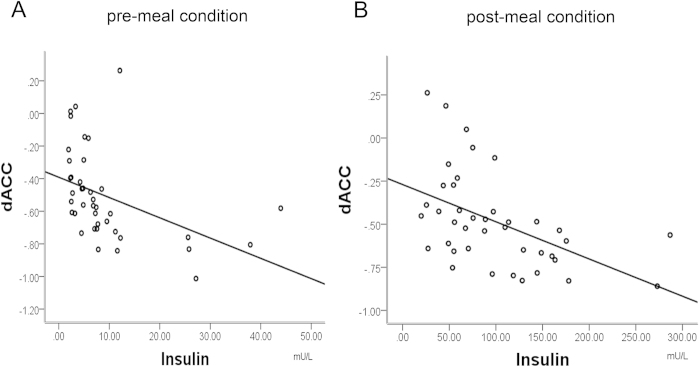
Correlation between the mean activity of dACC and plasma insulin in the pre-meal condition (part A) and the post-meal condition (part B).

We then selected regions of interest (ROIs) from these ALFF results (precunes and dACC), and correlated their mean activation levels with plasma insulin and subjective hunger ratings. A positive correlation between precuneus activation and subjects individual rating of hunger, adjusted for BMI, was revealed for conditions before liquid intake (r = 0.372, p < 0.036; Table 2 and Fig. 3). In addition, a significant negative correlation was found between dACC activation and plasma insulin before (r = −0.433, p < 0.005, Table 2 and Fig. 4A) and after liquid intake(r = −0.492, p < 0.001; Table 2 and Fig. 4B). Insulin resistance in terms of HOMA-IR paralleled the negative correlation with dACC ALFF before liquid intake (r = −0.361, p < 0.022), but not after liquid intake (p > 0.1).

## Discussion

In this fMRI study, we used a local metric of resting state activity to examine neural baseline activity in lean and obese men. Before and after liquid intake, obese men showed a significantly lower resting state activity in dACC, while a significantly greater activity in the precuneus was only found before liquid intake.

Our findings relate obesity to a misarrangement of neural systems involved in attention and baseline homeostasis. The precuneus belongs to the so called “default mode network” (DMN), which in its high baseline activation is thought to reflect a baseline state of brain function in the absence of external cognitive demands^15^. In our study, we found that the obese men had a significantly greater activity in the precuneus only in hunger state, not in satiety state. Previously it has been hypothesized that especially the precuneus is crucially involved DMN mediated self-referential thoughts^16^, rendering obese individuals in a hunger state to be more engaged in the processing information about their internal states^10^. Appetite control, a cognitive process downregulating ongoing internal focus on hunger or homeostatic regulation may be considered an antagonistic process related to task positive network activity^17^. As a result, especially during pre-meal condition, increased precuneus activity may thus be interpreted at the same time as a residual activity in the DMN as a result of insufficient activation of executive networks and as a representation of relatively increased self-focus and processing of subjective feeling of hunger. Consequently, our results showed a positive correlation between precuneus activity and the rating of hunger before food intake, directly mediating the behavioral relevance or the degree of subjective suffering from low energy supply. Consequently, their motivation to sustain such states and withhold from immediate food intake may be reduced compared to lean individuals. Our findings parallel those of Tataranni and colleagues, who reported greater activity in bilateral precuneus brain activity at hunger states compared to satiety^18^. Importantly, we did not find the precuneus to deviate in activity after food intake. Following a reward deficiency hypothesis, this would be counterintuitive in that especially after feeding, a deficient response in the reward system may lead to a sustained, if not increased group difference. In our study, every subject received a liquid meal in an amount proportional to their body size, which lead every subject to experience similar satiation after food intake. While we thus eliminated the possibility to provide less energetic supply to the obese subjects, a reward deficient processing mode would still expect reduced rewarding value for the same amount of relative caloric intake^19^. In our specific case, we could not find supporting evidence for an increased group difference after liquid intake, while this may however have been possible at different caloric levels. Under the circumstances of this experiment, one may suppose that suppression of food related thoughts was insufficient especially before feeding while this aspect was no longer relevant after the meal condition.

The dorsal ACC together with bilateral anterior insula belong to a “salience network” (SN), which forces the integration of highly relevant sensory stimuli^20^. Regions of the SN coactivate in response to metabolic stress, hunger and pleasurable feeling^21^. Previous fMRI studies found obese subjects showed increased activation in the insula and anterior cingulate cortex in response to food pictures, shown to induce reward anticipation^22^,^23^. However, so far it was not clear whether the intrinsic assignment of the SN may be a result of basic alterations in obesity which would also be present at resting state. In our study, we investigated the neural baseline activity at hunger and satiety states, and found generalized lower activity of dACC in obese men. This would potentially contradict the fact that during food anticipation, obese individuals were found to show hyper-responsivity in the SN which was thought to encode the reward value of food cues^4^. However, during both hunger and satiety states as investigated here, one may question explicit food anticipation, thus potentially explaining decreased activity in obese subjects. This would converge with structural findings of reductions in cortical thickness which were found as well in anterior cingulate cortex in obese adolescents^24^. This additional evidence of structural deficits would converge with reduced resting state activity, while it does not preclude this region to show increased activation during explicit tasks where cognitive processes themselves differ substantially between groups. In another study by Gautier and colleagues, obese individuals showed significantly decreased activity in the anterior cingulate cortex^25^. Increased or decreased activity may thus strongly depend on the actual contextual demands, while low baseline activations during rest might in principle also predispose to increased task related activations. Unfortunately, so far no study has investigated task related and resting state deviations in the same population.

While our findings seem to converge with previous work rendering ACC an important region with altered activity in obesity, its functional role seems to deviate from that of the precuneus. Its baseline aberration, irrespective of satiety further converged with its dependence from internal metabolic state, as best reflected by insulin measures. Previous work indicated that central nervous system (CNS) circuitry can be modified by insulin signals, which act as regulators of whole body energy homeostasis through their receptors in the CNS^26^. Insulin is crucially involved in regulation of appetite and food intake^27^ as its role is to relate physical as well as mental activity to the current metabolic supply. Gautier and colleagues found a negative relationship between changes in insulin and the activation of the orbitofrontal cortex (OFC) and bilateral precuneus following a liquid meal^18^,^25^. Our study showed plasma insulin concentrations of obese male subjects were significantly higher than in lean subjects before and after food intake. We also found a negative correlation between plasma insulin and the activation of dACC. Our findings thus may suggest that obesity and plasma insulin levels have substantial influence on the activation of dACC, supporting food intake via basic metabolic mechanisms and potentially independent from subjective motivation. Kullmann *et al.*, found task activation elicited by food stimuli differed between obese and lean individuals^8^, while in another study, quite consistent with our work, using an ICA based approach they found reduced connectivities in cingulo-opercular networks, overlapping with the salience network deficits described here as well as increased DMN connectivities in the location of bilateral precuneus^7^. Next to replicating their finding we could extend it to an observation of state dependence of such group effects. In another study, Kullmann *et al.*, found that intranasal insulin application in lean females induced increased activation in orbitofrontal cortex and hypothalamus, and they showed that after insulin application, a positive correlation between BMI and fractional ALFF (fALFF) was observed in dACC^28^. This indication of a relationship of fALFF increases after acute insulin application with BMI cannot be interpreted along the lines of our findings, where we rather find individuals with increased BMI to show reduced ALFF in the dACC with lowest values for subjects with highest insulin or HOMA-IR levels. Next to gender differences, our study and the study of Kullmann *et al.*, investigated chronic versus acute insulin effects unlikely to follow the identical mechanisms. No study has, to our knowledge, investigated the effects of intranasal insulin on dACC ALFF in obese males, while such study would be very helpful to disambiguate the diversity of findings related to insulin effects on brain activity in this region.

These observations should however be seen within some relevant limitations. Firstly, we did not estimate the palatability of the liquid meal in the subjects. Before the experiment, we estimated the palatability of the vanilla orally in all subjects suggesting that regular subjects favored the taste. We therefore decided to use this type of liquid meal in our experiment. However, we did not assess the degree of palatability using visual analogue scale, in which subjects would have rated their taste palatability for the vanilla flavor. Secondly, in order to reach to satiation, we only used the liquid formula meal, so there was no placebo condition (e.g. same capacity water) to account for changes in ALFF. Furthermore, we did not find differences in regions of the reward system. This may however have been the case for smaller caloric amounts, which may have resulted in different levels of reward system responses. Thirdly, we only used the male subjects in our study, not involved female. The study of Del Parigi *et al.* showed that sex-specific brain responses to a meal, and the differences in specific regions of the brain occurred against the similarities between the women and the men^29^. Furthermore, obesity in males and females may follow different routes, which may have an effect of BMI or insulin resistance^30^,^31^. In regard of the time dependent group effects in PCC/precuneus one should carefully note that such longitudinal changes are to be taken with caution given that there was no formal interaction of group and time in the exact location of the precuneus. Given then moderate sample size, it would have become problematic to sufficiently account for such potential interactions in our study. In our future study, we will enlarge the sample size and involve the female subjects. Fourthly, obesity has been associated with the alternation of cognitive and affective^32^,^33^. However, we did not perform any psychological tests or questionnaires. This may, in principle, affect the interpretation of the findings especially in dACC, which also plays an important role in executive functioning. Fifthly, when considering time by region interaction on the correlation of insulin and hunger ratings on dACC and precuneus activity, one needs to carefully consider that no information on a suited post-meal cluster for precuneus can be chosen, in contrast to dACC, which showed significant differences at both time points. The non-significance of correlation between hunger rating and precuneus activity thus may not fully exclude long term association between these two dimensions. Furthermore, the HOMA-IR correlation only mirrored insulin correlations with dACC at preconditions, so these complex interactions will remain subject to future analyses focusing on the long term change of associations between the two factors and the two brain regions. At last, we did not monitor whether the subjects fall asleep during the scan using the special equipment. Our subjects had thus been strictly instructed to keep still and do not fall asleep. After the scan, a technician checked with each participant whether this was rally done or if subjects are unsure about having fallen asleep. To reduce the risk of falling asleep, the duration of scanning was set to 6 minutes.

## Conclusion

ALFF, a measure of local resting state activity may be a useful tool to investigate the mechanisms of obesity. Our results suggest that obesity can be characterized by robust alterations of brain activity even in the absence of explicit stimulation. While affection spans across both DMN and SN, the relationship to deviant food intake seemed to be quite different: dACC may be seen as a basic biological marker of aberrant insulin related metabolic state in obesity, abnormal resting state in precuneus could be related more directly with increased subjective motivation towards increased eating behavior especially in the situation of hunger.

## Methods

### Subjects

In the present study, 20 lean men and 20 obese men were recruited via poster advertisement. Lean subjects were required to have a BMI from 18.5 to 23.9 kg/m^2^, and obese subjects were required to have a BMI > 28 kg/m^2^ using the adjusted Chinese guideline, which is an equivalent of WHO class I obesity^34^. All of subjects were right-handed and non-smokers. They had no history of illicit drug dependence or alcohol abuse and were not currently dieting to lose weight.

All participants gave written informed consent. This study was approved by the institutional review board of the Tianjin medical university. The methods were carried out in accordance with the approved guidelines.

All subjects completed the paradigm between 5:30 PM and 8:00 PM. On the day of the scan, subjects were fasted 6–8 hours prior to scanning. After lunch, subjects were asked not to ingest anything except water until the beginning of the experiment. In order to reach to satiation, a liquid formula meal (55% carbohydrate, 30% fat, 15% protein; Ensure-Plus 1.5 kcal/ml) was administered orally. The flavor of the liquid meal was vanilla. Every subject received a liquid meal in an amount proportional to their body size, which provided 40% of the personal resting energy expenditure^25^,^35^. Resting energy expenditure was assessed by indirect calorimetric analysis (Vmax Encore, Sensormedics, United States).

We assessed the degree of hunger using Visual Analogue Scales (VAS), in which subjects were asked to rate their sensations of hunger, scaled from 0 (‘not at all hungry’) to 100 (‘very hungry’). The assessment was performed before scanning in both pre-meal and post-meal conditions.

### Data Acquisition

The fMRI involved two sessions, before and after the liquid meal. Brain imaging data were acquired with a 3 Telsa MR imaging system (Signa-HDX, General Electric, United States) with an echo-planar imaging sequence: repetition time/echo time (TR/TE) = 2000/30 ms; flip angle 90°; slice thickness 4 mm (no slice gap); matrix 64 × 64; FOV 240 × 240 mm^2^, and voxel size 3.75 × 3.75 × 5 mm^3^. Each brain volume was comprised of 40 axial slices, and each functional run contained 180 image volumes, resulting in a total scan time of 360 s. All participants were instructed not to focus their thoughts on anything in particular, to keep their eyes closed and not to fall asleep during the MR acquisition. After the scan, a technician checked with each participant whether this was really done and keep every subject in our study complied with these instructions.

Blood samples were drawn before every scan session, and taken from cubital vein. Plasma glucose concentrations were determined by an automated clinical chemistry analyzer (Medical Cooperation, USA) and plasma insulin concentrations by a chemiluminescence immunoassay (CLIA) (Siemens Diagnostics, USA). Based on the blood samples before the food intake, homeostasis model assessment of insulin resistance (HOMA-IR) was calculated as HOMA-IR = glucose (mmol/L) × insulin (mU/L)/22.5. In this study, we acquired the data for the satiety sate at 30 minutes after liquid intake, which reported to reach the peak levels^36^,^37^.

### Data Preprocessing

The resting-state fMRI data were preprocessed using the Statistical Parametric Mapping software (SPM8, http://www.fil.ion.ucl.ac.uk/spm). The first 10 volumes for each subject were discarded to allow the signal to reach equilibrium and the participants to adapt to the scanning noise. The remaining 170 volumes were then corrected for the acquisition time delay between slices. All subjects’ fMRI data were within the defined motion thresholds (translational or rotational motion parameters lower than 2mm or 2°). We also calculated frame wise displacement (FD) to reflect the mismatch of volume to volume in head position^38^. The FD was obtained from the derivatives of the rigid-body realignment estimates. Several nuisance covariates (six motion parameters and their first time derivatives, the average signals of the whole brain, the average signals of ventricular and white matter, and the time points that had spike motion with FD > 0.5) were regressed out from the data. Next, subject specific structural image were segmented and normalized to Montreal Neurological Institute (MNI) space using a high-level non-linear warping algorithm named diffeomorphic anatomical registration using exponentiated lie algebra^39^. The same transformation parameters were then applied to the filtered functional images. The derived displacement was applied to individual mean realigned functional images, which was firstly linearly coregistered with corresponding structural images. The spatially normalized functional images were then resampled to 3 × 3 × 3 mm^3^ voxels. After normalization, images were smoothed using a Gaussian kernel of 8 × 8 × 8 mm^3^ full-width at half-maximum.

### ALFF calculation

Amplitude of low-frequency fluctuations maps were calculated using Resting-State fMRI Data Analysis Toolkit (REST, http://www.restfmri.net). The time courses are converted to the frequency domain using a Fast Fourier Transform (FFT). The square root of the power spectrum is calculated and then averaged across 0.01–0.08 Hz at each voxel, given that the averaged square root is taken as the ALFF^13^,^14^. The ALFF maps were then divided by whole brain mean ALFF values to normalize the global effects.

### Statistical analyses

ANOVA was calculated for the factors “group” and “feeding condition” using full factorial design in SPM8. The second-level two-samples *t* test was performed on the preprocessed data to test the group differences between healthy and obese male subjects with age as nuisance covariate before and after the liquid meal^40^. A correction for multiple comparisons was performed using a Monte Carlo simulation, resulting in a corrected threshold of *P* < 0.05 (AlphaSim program in REST), single voxel *P* = 0.001, 5000 simulations, FWHM = 8 mm, cluster connection radius r = 5 mm.

We then selected regions of interest (ROIs) from these ALFF results: dACC and precuneus.

Before and after liquid intake states, the ROIs of dACC were based on the mean activity of the voxels identified in the two-samples *t* test. After liquid intake, no precuneus difference in activity could be detected, so we only selected ROI of precuneus before liquid intake state. We then entered ROIs mean activation levels into correlation analyses with plasma insulin in both before and after liquid intake. And we entered ROIs mean activity levels into partial correlation analyses corrected for BMI with subjective hunger ratings. Kullmann *et al.* found the feeling of hunger modulated cognitive networks, independent of BMI^8^. So we excluded the BMI component and investigate partial relationships for ROIs and hunger ratings, which was seen in its result of the contribution of the hunger rating on the activation.

## Additional Information

**How to cite this article**: Zhang, B. *et al.* Altered baseline brain activity differentiates regional mechanisms subserving biological and psychological alterations in obese men. *Sci. Rep.* **5**, 11563; doi: 10.1038/srep11563 (2015).

## Supplementary Material

Supplementary Information

## Figures and Tables

**Table 1:** Characteristics of the study population.

	Lean (n = 20)	Obese (n = 20)	Group effect *P* value
Age (y)	20~28	20~28	
Body weight (kg)	63.52 ± 5.66	100.51 ± 13.32	0.015
BMI (kg/m^2^)	21.48 ± 1.43 (range: 18.5–23.9)	33.56 ± 3.53 (range: 28.0–41.5)	0.004
REE (kcal)	1627.25 ± 175.77	2331.5 ± 360.80	0.003
Glucose (mmol/L)			
Fasting	4.46 ± 0.44	4.12 ± 0.72	0.142
Postmeal	8.58 ± 1.86	6.84 ± 1.64	0.450
Insulin (uU/mL)			
Fasting	4.84 ± 5.30	14.81 ± 11.32	0.001
Postmeal	58.51 ± 27.13	143.12 ± 67.74	0.002
Hunger Ratings (mm)			
Fasting	71.56 ± 9.61	72.92 ± 13.24	0.330
Postmeal	20.63 ± 12.89	17.06 ± 12.99	0.738
HOMA-IR	0.80 ± 0.47	2.87 ± 2.96	0.006

**Table 2:** Results of the correlation between dACC and precuneus * mean activity and the rate of hunger and plasma insulin.

		hunger rating		*p*	Insulin		*p*
		pre-meal	post-meal		pre-meal	post-meal	
dACC	pre-meal	−0.066	—	0.719	**−0.433**	—	**0.005**
	post-meal	—	0.189	0.299	—	**−0.492**	**0.001**
precuneus	pre-meal	**0.372**	—	**0.036**	0.131	—	0.419
	post-meal	—	−0.062*	0.734	—	0.186	0.249

^*^Since no difference of ALFF was found in precuneus after liquid intake, this correlation was calculated with the ALFF values within the same region which showed significant group difference in pre-meal.
