# Supplementary material for: Altered baseline brain activity differentiates regional mechanisms subserving biological and psychological alterations in obese men
Source: Sci Rep. 2015 Jun 23;5:11563. doi: 10.1038/srep11563 (PMC4477369; doi:10.1038/srep11563)

**Altered baseline brain activity differentiates regional**

**mechanisms subserving biological and psychological alterations**

**in obese men**

**Bin Zhang, Derun Tian*, Chunshui Yu, Meng Li, Yufeng Zang, Yijun Liu, Martin Walter ***

Supplementary Figure 1. The results of main effect of group (in red, SI 1A) and feeding condition (in red, SI 1B) in the change of ALFF, revealed by the full factorial design in SPM8.


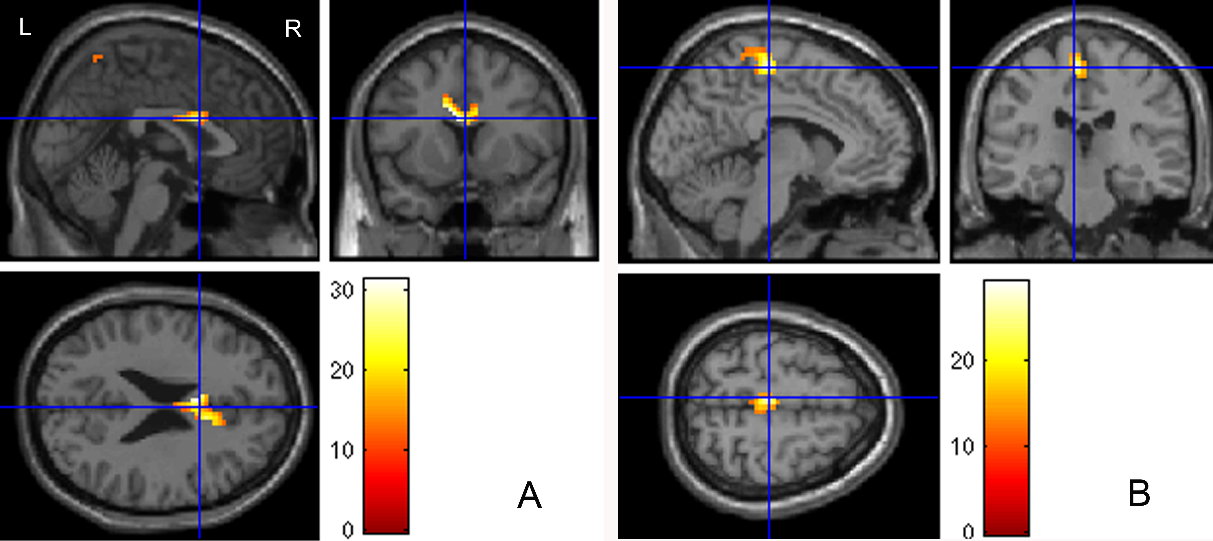


Supplementary Figure 2. The result of effect of the meal (ie before vs after) in group of healthy controls (in red, SI 2A) and obese subjects (in red, SI 2B).


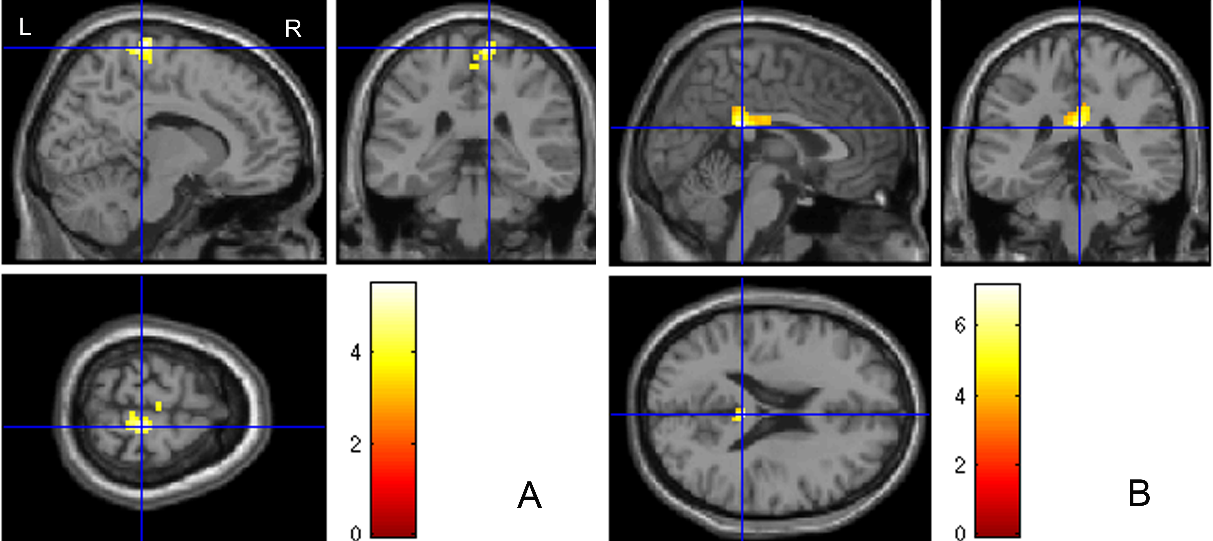

Supplement: Supplementary Information [file srep11563-s1.doc]
